# The association between continuity of care in the community and health outcomes: a population-based study

**DOI:** 10.1186/2045-4015-1-21

**Published:** 2012-05-23

**Authors:** Jacob Dreiher, Doron S Comaneshter, Yael Rosenbluth, Erez Battat, Haim Bitterman, Arnon D Cohen

**Affiliations:** 1 Chief Physician's Office, Clalit Health Services, Tel Aviv 62098, Israel; 2 Siaal Research Center, Division of Health in the Community, Ben-Gurion University of the Negev, Beer-Sheva 84105, Israel; 3 The Ruth and Bruce Rappaport Faculty of Medicine, Technion - Israel Institute of Technology, Haifa 32000, Israel

**Keywords:** Continuity of care, quality measures, healthcare services utilization, primary medicine, preventive medicine

## Abstract

**Background:**

The study goal was to assess indices of continuity of care in the primary care setting and their association with health outcomes and healthcare services utilization, given the reported importance of continuity regarding quality of care and healthcare utilization.

**Methods:**

The study included a random sample of enrollees from Clalit Health Services 19 years-of-age or older who visited their primary care clinic at least three times in 2009. Indices of continuity of care were computed, including the Usual Provider Index (UPC), Modified Modified Continuity Index (MMCI), Continuity of Care Index (COC), and Sequential Continuity (SECON). Quality measures of preventive medicine and healthcare services utilization and their costs were assessed as outcomes.

**Results:**

1,713 randomly sampled patients were included in the study (mean age: 48.9 ± 19.2, 42% males). Continuity of care indices were: UPC: 0.75; MMCI: 0.81; COC: 0.67; SECON: 0.70. After controlling for patient characteristics in a multivariate analysis, a statistically significant association was found between higher values of UPC, COC, and SECON and a decrease in the number and cost of ED visits. Higher MMCI values were associated with a greater number and higher costs of medical consultation visits. Continuity of care indices were associated with BMI measurements, and inversely associated with blood pressure measurements. No association was found with other quality indicators, e.g., screening tests for cancer.

**Conclusions:**

Several continuity of care indices were associated with decreased number and costs of ED visits. There were both positive and negative associations of continuity of care indices with different aspects of healthcare utilization. The relatively small effects of continuity might be due to the consistently high levels of continuity in Clalit Health Services.

## Background

Continuity of Care is a "core value" of patient care, especially in primary care medicine [1-8]. Continuity of Care is defined as consistent, "seamless" treatment over time involving various healthcare providers and settings. Continuity of care also refers to long-term care by a professional healthcare team, including effective communication for information sharing on such issues [1-8]. This paper explores the extent and impact of continuity of care in Clalit Health Services ("Clalit"), the largest healthcare provider organization in Israel.

Clalit operates both primary care clinics and hospitals, and provides all aspects of primary, secondary, and tertiary care to nearly 4 million enrollees (over 50% of the country's population). Some of the major principles on which Clalit has based its network of primary care clinics are the central role of the primary care provider in assuring high quality medical care for all members and the importance of a supplying a continuous source of primary care. The primary care physician is assumed to be the main pivot of the healthcare system. To illustrate this point, in 2009 Clalit's 4 million enrollees had 40 million contacts with primary care physicians, and only 9 million visits with consultants, 600,000 hospital admissions, and 900,000 ED visits that did not result in a hospital admission. Therefore, primary care visits consisted of 80% of all medical contacts in 2009. When referral to a consultant is needed, the patient is either referred by the physician or can be self-referred in several specialties (e.g., dermatology, orthopedics, ophthalmology). Primary care visits do not require co-payment, but visits to a consultant require a small co-payment (about US $6 for an unlimited number of visits within a calendar quarter). About 16% of clinics are solo clinics, 7% are group practice (where the list of enrollees is shared by two or more physicians), and most (77%) are team clinics. The patient first selects one of Clalit's clinics and then, if it is a team clinic, is offered a choice of several physicians within the clinic. For each patient a regular physician is specified. The primary care clinic supplies both curative and preventive services. Some preventive services are only initiated by physicians while other can be initiated by the nurse.

Since access to care and timeliness of making appointments is considered an important issue in the Israeli consumer's culture, even within Clalit breaks in continuity may occur when patients warrant encounters with another clinician. This may happen when patients need care outside working hours or in the absence of the regular physician due to illness, vacation, etc. Sometimes continuity breaks when patients choose to go to the emergency department (ED) without a referral.

Quality of care has been a major issue of interest for Clalit as a whole, but to date, it has been measured separately in primary care and in the hospital setting. Efforts are now underway to bridge that gap by developing quality measures dealing with continuity of care, both within the primary care setting and within the hospital, and in the interface between primary care and in the hospital setting, e.g., planned discharge, follow up with the primary care physician following admission for certain diagnoses, and processes where a good interface between primary and tertiary care is necessary to improve the quality of care (e.g., diagnosis and early treatment of cancer, follow-up for melanoma patients). As part of these efforts, we wanted to assess the association of published measures of continuity of care in the primary care setting in the general population, with a special focus on patterns of healthcare utilization.

Early attempts to define continuity of care were based on estimating the proportion of visits to a specific physician (longitudinal continuity). More recently, the literature has focused on issues such as care by the smallest number of professionals, continuity of data shared by caretakers (information continuity), good communications between caretakers working in a team or between various providers (team continuity), consistent approach to patient care management by all parties involved (management continuity), and the ongoing relationship between patients and care providers (relational/interpersonal continuity) [1-8]. The three major facets of continuity of care (longitudinal continuity, patient-professional relationship, and coordinated care) are related yet distinct concepts and therefore should be measured separately [1-14].

In primary medicine, continuity of care is typically defined as an ongoing relationship between a single caretaker and a patient beyond specific episodes of illness. Another way to think of continuity of care is to liken it to a loyalty contract between the patient and the person who has clinical authority on behalf of the healthcare service provider. This relationship, which may also be defined as longitudinal continuity, a "caring" relationship, or personal continuity, encourages improved communications, trust, and a sense of continuous responsibility. In family medicine, continuity of care differs from coordinated care, although continuity also improves coordination [5,7-9,13-19].

There is evidence that continuity of care is related to a high degree of patient satisfaction. Specific evidence associates continuity of care and aspects of healthcare services utilization. Continuity of care is important for specific categories of patients including women, the elderly, patients with chronic conditions, patients who consume many medications, individuals with limited social support networks (for whom the caretaker constitutes their main source of support), individuals with low educational attainment, and, in the United States, individuals insured by Medicare or Medicaid. Nonetheless, the significance of continuity of care attributed to specific patient groups varies, and many patients attribute greater weight to access to care rather than to continuity [6,15,18,20,21].

A patient-caretaker relationship in which a high level of continuity of care exists is characterized by improved patient-physician relations, including trust-building, mutual understanding, effective communications, a sense of responsibility over time [3,6,15,16,22-24], and better quality of care, including better identification of issues and diagnostic accuracy [3,6,15,16,19,22-26]. Continuity of care has also been associated with better management of patients with chronic conditions and maternity care outcomes, higher rates of compliance to medications, performance of screening tests, receipt of preventive medicine services and follow-up visits, and a reduction in hospitalizations, repeat hospitalizations, emergency department (ED) visits, and duration of hospitalizations [3,6,12,15,16,19,22-26]. This was especially true for older cardiac patients, patients with asthma, patients with diabetes, and hospitalizations due to chronic conditions. In contrast, no reduction in hospitalizations due to acute conditions was found [25].

In a previous study [27], increased continuity of care was associated with a statistically significant 44% reduction in the risk of all-cause hospitalizations, after controlling for patient case-mix, number of visits, and demographics. A statistically significant 46% reduction in hospitalizations due to chronic conditions was also found, while no reduction in hospitalizations due to acute illnesses was reported [27].

A correlation was also found with reduced healthcare expenses [12,17,25], especially as a result of reduced hospitalization rates, ED visits, clinic visits, and non-attendance rates [12]. A study of 4,000 patients in Belgium found that total healthcare costs for patients who were treated by a single physician were significantly lower than for patients who visited more than one physician in the two-year period preceding the study, after controlling for patient demographics and factors such as internal locus of control, physical functioning, mental functioning, co-morbidity, and number of routine visits to a clinic [18]. Correlations were also found with measures of patients' quality of life, alleviation of symptoms and chances of recurrence, such as time to return to a regular work schedule for patients with lower back pain [6].

Continuity of care also has potential shortcomings, as being treated only by a specific caretaker may reduce patients' ability to rapidly access an available caretaker in an emergency. Alternatively, visiting several caretakers may allow peers to check diagnoses or suggest additional possible directions for diagnostic explorations. Caretakers with specialties in various fields may complement each other. Higher continuity of care may paradoxically impair communications between the patient and the provider since their prior familiarity may reduce the duration of each visit and prevent patients from raising new issues. Nonetheless, a comprehensive literature review of this topic did not find evidence of damage caused by higher continuity of care [25].

Since continuity of care is a multi-faceted concept, its assessment requires several measures [3,20,28-36]. Most indices of continuity of care address the temporal aspects of patient-caretaker interactions, such as duration of care, frequency of interactions, concentrated vs. distributed care among several caretakers, and sequence of care [3,20].

The aim of this study was to describe selected measures of various aspects of continuity of care (concentration of care, distribution among several caretakers, short-term sequence) within the primary care setting and examine their association with healthcare services utilization, including hospitalizations, ED visits, and duration of hospitalizations, and preventive medicine quality indicators reflecting the quality of preventive services (performance of screening tests) in a sample of the general adult population of Clalit. While the subject has been previously investigated in other health care systems [5,6,8,10,12,13,15-17,19,22,23,25,36] it has not been previously studied in Israel. For this study, we utilized Clalit's extensive database, which includes demographic and clinical information, including utilization of healthcare services, thus facilitating studies like the present one.

## Methods

The present study was based on retrospective data of members of Clalit for 2009. The Clalit database includes 4,000,000 enrollees. Sampling the entire database is feasible using the last digit of the ID number and/or the two digits before the last one. As It is technically non-feasible to run queries on a population of that size, we combined these two methods to yield about 4,000 enrollees, of whom 1,713 met our inclusion criteria - patients aged 19 years or older, who visited their primary care physician at least three times in 2009, from a population of 2,649,870 enrollees aged 19 years or older.

The main goal of the study was to identify associations of continuity of care indices with healthcare utilization pattern by a logistic regression model. We estimated that up to 20 variables are likely to be included. Since the rule of thumb is to include at least 15 observations per parameter, at least 300 patients would have to be included in the analysis to yield significant results [37]. Therefore, the sample size available for analysis (1,713 patients) was satisfactory. The cutoff of 3 visits was necessary because continuity of care is always perfect for patients with one visit, and even among patients with two visits, values of indices could shift from 0 to 1 with minute changes in the patterns of visits. Patients treated at a group-practice clinic were excluded, because in such clinics more than one physician is the regular source of care for the patient and the individual physician that participated in the clinical encounter could not be identified. These patients comprised 8% of Clalit's enrollees in 2009 (7% of clinics). No other exclusions were made. Pregnant women were included in the analysis.

Variables used in this study were derived from Clalit's computerized databases. Clalit maintains a comprehensive database that includes demographic information, utilization of primary and consultative medicine services, laboratory tests and imaging, ED visits, hospitalizations, chronic diagnoses, medications, and primary medicine quality measures. The accuracy of Clalit's database for chronic diagnoses has been previously reported to be high [38]. Almost all Clalit members have a single regular physician. For each visit, data include date of visit and type of visit (ordinary visit, house call, telephone call, visit without the patient's presence [visits for renewing prescriptions or issuing medical documents for the patient], visits for administrative reasons, and unknown/undefined type of visit.). The current study included ordinary visits and house calls only. Visits to the nurses' room only were not included in the present analysis.

The following four continuity of care measures were computed for each patient, based on formulas described in the literature [3,9,20,25] (see Appendix 1 for formulas and illustrative examples):

### Usual Provider Continuity (UPC)

This index describes the proportion of visits to the patient's regular physician out of all visits. It ranges from 0 (no visit to the regular physician) to 1 (all visits made to the regular physician). Since all Clalit enrollees have a regular physician, the UPC was calculated according to the above definition (see Appendix 1). According to the literature, if no regular physician is defined for a patient, the index is computed for the physician the patient visited most frequently [3,9,20].

### Modified Modified Continuity Index (MMCI)

This index focuses on the dispersion between providers and is based on the number of caretakers and number of visits only. Index values range from 0 (each visit made to a different physician) to 1 (all visits made to a single physician). The use of this index in research has become widespread in recent years [3,25].

### Continuity of Care index (COC)

This index weights both the frequency of visits to each caretaker and the dispersion of visits between caretakers. Index values range from 0 (each visit made to a different physician) to 1 (all visits made to a single physician) [3,9,20].

### Sequential Continuity Index (SECON)

This index measures the number of visits made to the caretaker whom the patient saw in the most recent visit. This index is useful for assessing the need to share information among caretakers. Index values range from 0 (every visit made to a physician other than the physician seen in the previous visit) to 1 (all visits made to a single physician) [3,9].

While the UPC focuses on the proportion of visits to the main provider, and does not consider the dispersion among other providers, the MMCI focuses on the dispersion between providers, and the COC is a combined measure that weights these two aspects into a single metric. The SECON is related to the short-term aspects of continuity, rather than the long-term (see Appendix 1).

Additional independent variables included demographics (sex, age, marital status, and country of birth), clinical variables (underlying chronic conditions, including smoking, obesity, and hyperlipidemia), Charlson's comorbidity index [39], and features of the primary clinic including ethnicity of the main population served by the clinic (i.e., Jewish/Arab) and the socioeconomic score of the clinic's area. The socioeconomic score was available at the clinic level, and taken from the socioeconomic status of the relevant census tract from the Israel Central Bureau of Statistics' database.

The dependent variables (the primary study outcomes) included utilization of healthcare services and their costs, and several quality measures of preventive medicine used in Clalit. Healthcare services utilization data refer to 2009, and included the number of hospitalizations; total number of days and cost of hospitalizations; the number of visits to the ED, hospital outpatient clinics, and community consultative medical clinics, and the cost of these visits; purchase of medications; and preventive medicine quality measures. Out of the extensive list of 67 quality indicators used in Clalit for evaluating primary care, several indicators were chosen for the analysis, including recording smoking status, blood pressure measurement, height and weight measurements, renal function screening, and cancer screening tests (occult fecal blood test and mammography). These indicators were chosen because they relate to preventive services intended for a large target population (all individuals within an age or sex group, and not just those with chronic diseases or special care problems).

## Statistical Analysis

Continuity of care indices were analyzed as both continuous and dichotomous variables, based on two possible reference points (the median and lowest quartile). The correlations between the four continuity of care indices and healthcare services utilization data were calculated using Spearman's rank correlation coefficient. For preventive medicine quality measures, correlations were tested using the Mann-Whitney test for median comparison.

We constructed multivariate models to test the adjusted effect of each of the continuity of care indices on the extent of healthcare services utilization and on preventive medicine quality of care indices. In these models, utilization of healthcare services was predicted using a linear regression model, and compliance with preventive medicine quality measures was tested separately for each continuity of care index using a logistic regression that included the clinical and socio-economic indices described above as confounders. Goodness of fit of the models was assessed by computing the rate of explained variance (R^2^) and C statistic of each model. Statistical significance was determined at the 0.05 level; statistical analyses were performed using SPSS for Windows software, Version 17.0.

The study was approved by the Institutional Review Board of the Meir Medical Center, which is responsible for community-based studies conducted in Clalit.

All authors declare that they have no competing interests.

## Results

Out of a population of 2,649,870 adult enrollees, 12% had no visit with a primary care physician, 12% had a single visit, 11% had two visits, and 65% visited the primary care clinic three times or more. A random sample of 1,713 patients who visited their primary care physician at least three times in 2009 was selected for the study (Table 1). Within this sample, the majority of patients were female, one-quarter were 65 or older (mean age: 48.9, range: 19 to 97). The sample comprised a greater number of females, individuals age 65 or older, and unmarried individuals, compared to the target population, i.e., all Clalit members. No difference was found between the sample and the target population in terms of the socio-economic clinic scores (Table 1). Seventy-nine percent of patients were treated by a salaried physician and the rest by self employed physicians. This proportion was similar to the percentage of salaried physicians in Clalit (about 80%).

**Table 1:** Demographic features of patients included in the sample (N = 1,713).

Variable	Category	Number of participants (% of sample) N = 1,713	Number of participants (% of general population)	*p *value
Sex	Male	721 (42.1%)	1,261,204 (47.6%)	< 0.001
	Female	992 (57.9%)	1,388,666 (52.4%)	
Age group	< 65 years	1,293 (75.5%)	2,149,186 (81.1%)	< 0.001
	≤65 years	420 (24.5%)	500.720 (18.9%)	
Marital status	Married	1,072 (62.6%)	1,575,082 (59.4%)	0.008
	Unmarried	641 (37.4%)	1,074,825 (40.6%)	
Socio-economic score of the clinic	Low	707 (41.8%)	1,087,160 (41.8%)	0.711
	Medium	659 (39.0%)	995,797 (38.3%)	
	High	325 (19.2%)	519,387 (20.0%)	
Sector of the clinic	Arab	319 (18.6%)	539,006 (20.3%)	0.114
	Ultra-Orthodox	43 (2.5%)	76,725 (2.9%)	
	General	1,351 (78.9%)	2.034.176 (76.8%)	

**Figure 1:**
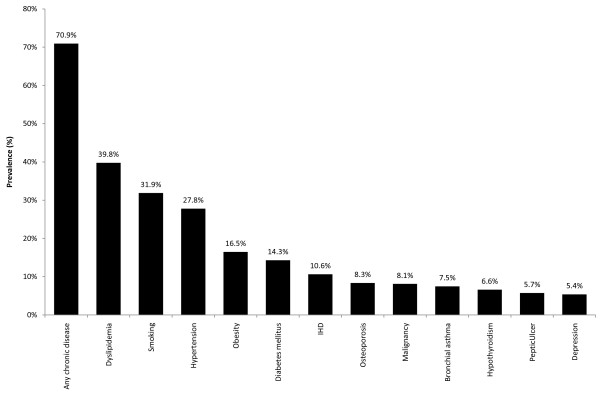
**Prevalence of chronic and risk conditions in the sample (N = 1,713)**.

The median number of visits to a primary physician was 6 (3-57) and the median number of caretakers per patient was 2 (1-11). Most participants (70.9%) had at least one underlying chronic condition. Figure 1 illustrates the distribution of the main chronic conditions (those with a prevalence of over 5%). The most frequent underlying illnesses were hyperlipidemia (39.8%), hypertension (27.8%), diabetes (14.3%), and ischemic heart disease (10.6%).

The four selected continuity of care indices and data regarding services utilization in 2009 are described in Table 2. Continuity of care indices were: UPC: 0.75 ± 0.25; MMCI: 0.81 ± 0.21; COC: 0.67 ± 0.30; SECON: 0.70 ± 0.31. 36.1% of the participants experience "perfect" continuity of care (computed value of 1.0 on all measures). The median values of the four indices range from 0.67 to 0.86 (Table 2). The four indices were highly correlated with each other. Spearman's rho values ranged from 0.935 to 0.996.

**Table 2:** Stratified analysis of continuity of care indices by demographic features (N = 1,713).

		N	UPC	MMCI	COC	SECON
			Mean (Median)	Mean (Median)	Mean (Median)	Mean (Median)
All patients		1,713	0.76 (0.81)	0.81 (0.86)	0.67 (0.68)	0.70 (0.75)
Sex	Male	721	0.76 (0.83)	0.80 (0.86)	0.67 (0.71)	0.69 (0.75)
	Female	992	0.75 (0.80)	0.82 (0.86)	0.67 (0.67)	0.70 (0.75)
	*p *value		0.741	0.252	0.719	0.569
Age (years)	19-40	684	0.70 (0.71)	0.76 (0.80)	0.61 (0.55)	0.63 (0.67)
	41-60	500	0.77 (0.83)	0.82 (0.88)	0.68 (0.70)	0.71 (0.75)
	61-97	529	0.82 (0.89)	0.87 (0.91)	0.75 (0.80)	0.76 (0.81)
	*p *value		< 0.001	< 0.001	< 0.001	< 0.001
Region	South	190	0.71 (0.73)	0.78 (0.80)	0.61 (0.55)	0.66 (0.67)
	Center	1,010	0.76 (0.83)	0.81 (0.87)	0.68 (0.70)	0.70 (0.75)
	North	513	0.77 (0.83)	0.82 (0.88)	0.69 (0.71)	0.71 (0.75)
	*p *value		0.024	0.138	0.010	0.161
Charlson's comorbidity index (without age)	0	883	0.73 (0.75)	0.79 (0.83)	0.65 (0.61)	0.67 (0.70)
	1	395	0.76 (0.83)	0.82 (0.88)	0.68 (0.72)	0.71 (0.77)
	2+	415	0.80 (0.86)	0.86 (0.89)	0.72 (0.75)	0.74 (0.78)
	*p *value		< 0.001	< 0.001	0.001	0.001
Physician	Salaried	1,345	0.75 (0.80)	0.81 (0.86)	0.67 (0.67)	0.69 (0.75)
	Self-employed	368	0.76 (0.86)	0.83 (0.90)	0.69 (0.75)	0.70 (0.80)
	*p *value		0.513	0.142	0.158	0.602

Regarding healthcare services utilization, 19.4% of the participants visited the ED at least once during 2009 and 75.1% made at least one consultation visit to a specialist during the year. At least one hospitalization was experienced by 14.7% of the participants. 52.5% of participants visited an outpatient clinic at least once during the year.

Subgroup analysis of the continuity of care indices by patient characteristics is outlined in Table 2. All continuity of care indices increased with increasing age and severity of comorbidity as assessed by Charlson's comorbidity index. UPC and COC were lower in southern Israel. No differences were found between males and females and between salaried and self-employed physicians (Table 2).

An analysis of the correlations between the four indices of continuity of care and various aspects of healthcare services utilization indicated weak, albeit statistically significant, correlations (Table 3). In a univariate analysis, higher continuity of care was associated with a greater number of visits to consultative physicians and outpatient clinics and their costs, and the cost of medications. In contrast, a higher degree of continuity of care was found to be inversely related to the number of visits to ED facilities and to the total costs of such visits.

**Table 3:** Correlations* between continuity of care indices and healthcare services utilization (scope and costs).

Outcome	UPC	MMCI	COC	SECON
No. of ED visits	- 0.087*	- 0.081*	- 0.094*	- 0.099*

Cost of ED visits	0.086*	- 0.077*	- 0.092*	- 0.096*

No. of outpatient clinic visits	0.042	0.080*	0.044	0.032

Cost of outpatient clinic visits	0.036	0.071*	0.036	0.024

No. of hospitalizations	0.028	0.042	0.028	0.020

No. of hospitalization days	0.031	0.045	0.031	0.023

Cost of hospitalization	0.033	0.047	0.033	0.025

No. of medical consultation visits	0.033	0.062*	0.032	0.038

Cost of medical consultation visits	0.040	0.062*	0.038	0.039

Cost of medications (to the patient)	0.119*	0.159*	0.122*	0.113*

Cost of medications (to the HMO)	0.009	- 0.001	0.008	0.007

(*) Correlations between continuity of care indices and healthcare outcomes and costs are statistically significant (*p *< 0.05).

Similar correlations between continuity of care indices and healthcare services utilization and costs were found in comparisons between low (bottom quartile) and middle-high levels of continuity of care (remaining quartiles). The cut-off points of the bottom quartiles were 0.55 for UPC, 0.68 for MMCI, 0.40 for COC, and 0.50 for SECON.

Patients with higher continuity of care were more likely to have documented weight and height measurements than patients with lower continuity of care. A similar correlation was found for recording participants' smoking status, although it was not statistically significant. In contrast, an inverse correlation was found for hypertension measurement records. No statistically significant correlations were found between continuity of care indices and performance of cancer screening tests (occult fecal blood tests and mammography).

Multivariate models that included a single continuity of care index and several confounding variables (excluding the number of visits and providers) indicated a statistically significant effect for several indices of continuity of care on healthcare utilization outcomes (Table 4). Higher continuity of care was associated with a decreased number of ED visits and their costs, and increased costs of medical consultation visits, after controlling for sex, age, ethnicity (Arab vs. Jewish), marital status (married vs. all other statuses), type of residential area (urban vs. rural), socio-economic score of the clinic (high, intermediate, and low), and selected underlying chronic conditions (which were different for different models; Table 4). The regression coefficients of 0.13-0.18 associated with the number of ED visits can be translated into a 6-8% decrease in the mean annual number of ED visits for every 0.1 increase in the continuity indices. Charlson's comorbidity index was not included in the model because including specific types of comorbidity was associated with a better fit of the model. No other parameters of healthcare utilization were found to be significantly associated with continuity of care indices, including the number of hospitalizations; total number of days and cost of hospitalizations; hospital outpatient clinics, and purchase of medications. Similar results were found when continuity of care indices were dichotomized (lower quartile vs. all other quartiles) prior to inclusion in the multivariate models (data not shown).

**Table 4:** Selected linear regression models for health outcomes.

Explanatory variables (continuity of care indices)	B	Confidence interval (95%)		*p *value
			
		Lower	Upper	
**Number of ED visits**				

**UPC**	**0.16 -**	**0.29 -**	**0.03 -**	**0.019**

MMCI	0.13 -	0.29 -	0.03	0.116

**COC**	**0.18 -**	**0.29 -**	**0.07 -**	**0.001**

**SECON**	**0.16 -**	**0.27 -**	**0.05 -**	**0.004**

**Costs of ED visits**				

**UPC**	**- 82**	**150 -**	**14 -**	**0.018**

MMCI	60 -	142 -	21	0.148

**COC**	**91 -**	**148 -**	**35 -**	**0.002**

**SECON**	**81 -**	**138 -**	**25 -**	**0.001**

**Costs of medical consultation visits**				

**UPC**	22	16 -	59	0.254

**MMCI**	**49**	**5**	**94**	**0.030**

COC	10	22 -	41	0.545

**SECON**	18	13 -	49	0.260

Results are adjusted for sex, age, ethnic origin, marital status, residential area (urban vs. rural), socio-economic score of clinic, and selected underlying chronic conditions.

Regarding quality measures related to preventive medicine, continuity of care indices were only associated with hypertension measurement records (C statistic = 0.82), although the correlation did not reach statistical significance after adjustment for confounders.

## Discussion

Clalit attributes great significance to monitoring the health status of its members over time. Its computerized databases make it possible to conduct in-depth studies of the continuity of care experience of members and the organization's ability to maintain high standards of continuity of care over time. The current study illustrates the significance of this concept, and presents preliminary empirical findings on the association of continuity of care indices with utilization of healthcare services and quality measures related to preventive medicine. A statistically significant correlation was found between higher values of continuity of care indices and a decreased number of ED visits and their costs, after controlling for participants' background variables. In contrast, higher values of MMCI were associated with a higher cost of consultative medicine.

Given the inclusion criteria, the difference between the study population (patients visiting their primary care physician at least 3 times a year) and the general population should be taken into account. The study population was older, more likely to be female and married, and had a higher prevalence of certain chronic diseases (e.g., diabetes) than the general Israeli population. These findings are not surprising, as patients frequently visiting the clinic are *a priori *more likely to be older, sicker, more likely to be female, and less likely to be single,

In this study, continuity of care indices were relatively high (average values ranged from 0.67 to 0.81 for various indices; a value of 1 was computed for 36.1% of participants). Other studies found lower values of continuity of care indices. For example, in Delaware, United States, MMCI values were found to be between 0.48 and 0.51 [22]. COC values from 0.28 to 0.46 were reported in England [8,20], although UPC values were higher (0.50-0.68) [20]. Another study [16] found higher values of UPC (0.79 in the USA and 0.72 in England), similar to the values found in the current study. The high continuity of care in Clalit could be explained by Clalit's policy of assigning a regular physician to virtually all patients, an on-going relationship with the primary care physician, sometimes for many years, and a strong commitment to patients' satisfaction with the quality of service, monitored periodically by surveys. Further steps that could increase continuity of care could be routinely recommending that the patient maintain continuity of care with the regular physician when assigning a new appointment (rather than making the earliest possible appointment with another physician) and incorporating continuity indices into the National Program for Quality Indicators in the Community in Israel [40].

As described in the Background section, health outcomes previously reported as most strongly associated with continuity of care are an increased use of preventive medicine services and a reduced number of hospital admissions [3,6,15,16,19,22-26]. Other health outcomes reported include the quality of the patient-physician relations and communication, management of patients with chronic conditions, and patients' quality of life [6,12,17,25,27].

In the present study we found continuity of care to be associated with decreased utilization of the ED and increased utilization of ambulatory consultations. The association with decreased utilization of the ED is in accordance with the literature [3,6,12,15,16,19,22-26]. The increased number of referrals to consultants seen with increased continuity of care is paradoxical, and can be explained by (1) assuming that when patients are seen by a regular source of care, they are more likely to be referred to ambulatory care than to emergency services; (2) assuming that physicians with a continuous relationship with a patient might be more likely to refer that patient for consultation than a physician who hardly knows the patient, and (3) considering the fact that some consultations can be self-referred (see above), in which case they might be independent of the primary care physician's relations with the patient. However, the overall effect of all the continuity of care measurements was very small. Most *r *values detailed in Table 3 were less than 0.1. Therefore, the extent to which the variance can be explained (which is equal to r^2^) is less than 1% for most measurements. Even so, the regression coefficients of 0.13-0.18 associated with the number of ED visits actually mean that (given that the annual average number of ED visits of 0.225 per enrollee), each increase in 0.1 of the continuity index can be translated into a 6-8% decrease in the number of ED visits.

The lack of association with hospitalization is in contrast with our hypotheses and with previously published findings, and can be explained by the fact that the present study deals with a sample of a relatively healthy general population. Patterns of care in this population may be different than those of special populations such as chronically ill patients or the elderly. Furthermore, we included all types of hospitalizations (due to both acute illness and exacerbations of chronic disease states) in the present analysis. Acute, unavoidable hospitalizations may be less likely to be prevented by better continuity of care and might be more prevalent in a sample of a healthy general population. Thus the association between continuity of care and hospitalizations is less prominent than expected.

We found some correlation between continuity of care and quality measures related to preventive medicine, such as screening for smoking and measuring weight and height, but no association with cancer screening and an inverse association with screening for hypertension. It could be hypothesized that screening for cancer is dependent more on the patient's compliance and access to those services than to the nature of the physician-patient relationship, and therefore no association was found between continuity of care and cancer screening. As for the findings related to screening for hypertension, these are hard to explain and may reflect a chance finding. There might be a threshold effect, above which differences in continuity are unlikely to make a difference. Given the high overall continuity of care within Clalit, it is possible that the preset analysis was unable to relate small differences in continuity between the lowest quartile and the other three quartiles. Different findings might arise in a population with a lower continuity of care and greater dispersion.

Choosing a sample of patients with frequent visits to their family physician may explain the strongest univariate association between continuity of care indices in this study, the relation with prescription of drugs and also medication cost. On the other hand, choosing subjects based on visits to family physicians, and not nurses, might have reduced the ability to correctly reflect the performance of prevention tests and blood pressure measurements.

The four indices were highly correlated with each other. This could be explained by the relatively high values of these induces, with 36% of patients having a value of 1.0 for all four parameters and median values ranging from 0.68 to 0.86. Despite this, we recommend future studies to include all four indices, as each of them measures continuity of care in a different way and could behave differently in a population with a lower level of continuity of care. Furthermore, since there are several different types of continuity (i.e., longitudinal continuity, informational continuity, relational continuity, etc., as described in the Background section), the relationship between continuity of care and positive outcomes is complex. Future studies should probably dissect the overall concept into a series of measures.

Correlations between continuity of care and improved health outcomes such as fewer ED visits or improved compliance with preventive medicine instructions do not necessarily constitute evidence of a causal relationship. A relationship in the opposite direction is also possible -- Salutz and Lochner [12] suggested that patients with better health outcomes may be more satisfied with their physician and therefore return to see them. The correlation may also be explained by patients' individual characteristics that are directly related to improved outcomes. Furthermore, improved organization of healthcare services may also enhance patients' health outcomes and maintain continuity of care [12]. On the other hand, it can also be hypothesized that patients with better health outcome have a smaller number of visits to the primary care clinic, so that mathematically, a single visit to a physician other than the regular provider could have a great influence on decreasing continuity of care indices. This could explain the association between higher Charlson comorbidity index and better continuity of care found in the present study.

The current study is unique in the selection of its sample, which is taken from all adult members of Clalit (aged 19 years and older) and includes individuals who enjoy good health alongside others who have one or more chronic condition of varying levels of severity. The findings of this study illustrate once again that continuity of care indices are associated with health outcomes and measures related to preventive medicine. The statistically significant correlations obtained indicate that continuity of care experienced by patients is a measure that is worthy of attention. Whether intentional use of continuity of care indices contributes to the prediction of healthcare services utilization and costs and to the prediction of patients' compliance with preventive medicine should be the subject of further study.

The current study has several limitations. Continuity of care indices were related to visits to family physicians only. Including visits to all physicians with whom patients consult might provide a different picture. The study is based on 2009 data only and the situation may be different in earlier or later years. Healthcare services utilization was measured concurrently with continuity of care, and it may be advisable to measure the correlation between continuity of care over several years and healthcare services utilization in the subsequent year. Clinic visits included in this study were visits that were marked as either ordinary visits or house calls. Although visits without the patient's presence were excluded from this study (typically these were visits by a family member who requested prescriptions or medical documents on the patient's behalf), it cannot be ruled out that family physicians marked visits without the patient's presence as ordinary visits. Furthermore, the study included visits that may have lacked a genuine therapeutic encounter, for example, when the patient came to renew a prescription or request various documents. Several preventive medicine quality measures (such as cancer screening) are relevant only for patients over age 50 and therefore the proportion of such patients in the sample may have been too small to generate statistically significant results; in the future, it might be useful to rerun those analyses using a larger sample size.

Since nurses are also an important source of referral for preventive services, focusing on visits to the primary care physician might have obscured our ability to detect the association with continuity of care. Given the inclusion criteria, the difference between the study population and the general population, including demographics and the prevalence of chronic diagnoses, should be taken into account. On the other hand, some chronic diseases (e.g., obesity) were probably under-diagnosed.

Health policy implications of this study stress the importance of monitoring and improving continuity of care within the primary care setting, although the associations found in the present study are not strong. Besides letting each organization work on its own to improve continuity, one might argue that given the importance of continuity, there should be transparent or published measures of it so that the Israeli public can know the performance of each of the HMOs. This is important because the continuity of care among the other HMOs is unknown and might demonstrate lower continuity with greater variability and stronger associations with healthcare utilization. It might be reasonable to focus quality indicators on specific subgroups of interest, such as the elderly, the chronically ill, or patients with increased healthcare utilization (e.g., repeated ED visits).

Several additional questions remain to be answered. Consultations in some specialties do not require referrals. However, not all patients are aware of this option, and some patients prefer to consult the primary care physician first even when they can directly go to a consultant. How does the pattern of utilization of consultations differ between those with and without referrals? Is the probability of referral to a medical consultant more likely for high vs. low users of consultations? Does continuity of care affect ED self-referrals differently (interrupting continuity of care) compared with primary care physicians' referrals? How does the association with continuity of care differ in avoidable vs. unavoidable admissions? How does the role the primary care physician (gate-keeper vs. a true source of advice) affect the association between continuity of care and utilization of consultations? Answering these questions could be the subject of future studies.

## Conclusion

In the present study, continuity of care indices was associated with a decreased number of ED visits and their costs, but with a higher cost of consultative medicine. In a follow-up study, we intend to examine the correlations between continuity of care indices and healthcare services utilization and outcomes in specific target populations, such as patients with chronic conditions, elderly patients, and patients lacking social support systems. In such a selected population, we might better assess the effect of continuity of care indices on healthcare services utilization, health outcomes, and preventive medicine quality measures. Continuity of care seems to be a multi-faceted issue and its components should be measured and improved separately.

## Appendixes

### Appendix 1: Formulas used to compute the selected continuity of care indices and illustrative examples

This appendix gives details of the formulas used to calculate the continuity of care indices. To illustrate the differences between indices, let us consider a patient who visits a clinic with 3 providers (A, B, and C) 8 times in a given year.

#### A. Usual Provider Continuity Index (UPC)

$$\text{UPC index}={\text{n}}_{\text{i}}/\text{N}$$

where n_i _is the number of visits to a regular physician by patient i, and N is the total number of patient i's visits to a physician. If visit patterns are used to determine "regular" providers, and no regular provider is defined, the following formula can be used:

$$\text{UPC}=\frac{\text{max}\left(n_{1},n_{2},....n_{k}\right)-1}{N-1}$$

where max(n_1,_n_2_, ... n_k_) is the number of visits to the provider with whom the patient had the greatest number of visits, and N is the total number of visits by the patient to all providers during the same period. If the sequence of visits was AAAABBBC, then the UPC is 4/8 = 0.50.

#### B. Modified Modified Continuity Index (MMCI)

$$MMCI=\frac{1-\frac{k}{N+0.1}}{1-\frac{1}{N+0.1}}$$

where k is the number of providers and N is the total number of visits to all providers in a given period. If the sequence of visits was AAAABBBC, then the MMCI is (1-3/8.1)/(1-1/8.1) = 0.72. If, for comparison, the sequence of visits was AAAABBBB, then the MMCI is (1-2/8,1)/(1-1/8.1) = 0.86, although both have the same UPC (0.5).

#### C. Continuity of Care Index (COC)

$$\text{COC}=\frac{\overset{k}{\underset{i=1}{\sum }}n_{i}^{2}-N}{N\left(N-1\right)}$$

where k is the number of providers, n_i _is the number of visits per provider I, and N is the total number of visits to all providers in a given period. If the sequence of visits is AAAABBBC then the COC is 0.32, while if the sequence of visits is AAAABBCC, the COC is 0.29, although both have the same UPC (0.50) and same MMCI (0.72).

#### D. Sequential Continuity Index (SECON)

$$\text{SECON}=\frac{\phi_{\text{i}}+...+\phi_{n-1}}{N-1}$$

where φ_i _takes a value of 1 if the current and subsequent visits are made to the same provider, and has a value of 0 if these visits are made to different providers. N is the total number of visits in the period. The final visit in the period is ignored and therefore the formula refers to N-1. If the sequence of visits was AAAABBBB, then the SECON is 6/7 = 0.86, while if the sequence of visits was ABABABBA, then the SECON is 1/7 = 0.14, although they both have the same values for UPC (0.50), MMCI (0.86) and COC (0.43).

## Abbreviations

COC: Continuity of Care Index; ED: Emergency department; MMCI: Modified Modified Continuity Index; SECON: Sequential Continuity; UPI: Usual Provider Index

## Competing interests

The authors declare that they have no competing interests.

## Authors' contributions

JD participated in the conception and design of the study, acquisition of data, analysis and interpretation of data, and drafting of the manuscript. DSC participated in the conception and design of the study, acquisition of data, analysis and interpretation of data, and drafting of the manuscript. YR aided in the literature review, conception and design of the study, and drafting of the manuscript. EB aided in the conception and design of the study, acquisition of data, analysis and interpretation of data, and drafting of the manuscript. HB participated in the conception and design of the study, and critically revising the manuscript for intellectual content. ADC participated in the conception and design of the study, analysis and interpretation of data, drafting of the manuscript, and critically revising the manuscript for intellectual content. All authors have given final approval of the submitted version.

## Authors' information

**Erez Battat **is an analyst and statistician with more than 10 years of experience at the research department of the Chief's Physician office, at Clalit Health Services. He holds a Master in Business Administration degree.

**Haim Bitterman **is the Chief Physician of Clalit Health Services and a Professor of Medicine in the Bruce and Ruth Rappaport Faculty of Medicine at the Technion - Israel Institute of Technology, Haifa. Formerly, he was the Chairman of Medicine in Carmel Medical Center in Haifa

**Doron S. Comaneshter **is an epidemiologist and statistician at Clalit Health Services with more than 10 years of experience in analyzing data. He is also a Ph.D. candidate at Haifa University, specializing in patients' rights and ethics issues concerning medical treatment.

**Arnon D. Cohen**, MD, MPH, PhD is the head of the Department of Quality Measurement and Research in the Chief Physician's Office at Clalit Health Services headquarters. Prof. Cohen is an associate professor at Ben-Gurion University. Prof. Cohen formerly served as the medical director of the southern Negev administration of Clalit Health Services.

**Jacob Dreiher **is a physician and epidemiologist, currently the director of Hospital Accreditation Department at the Hospital Division of Clalit Health Services. Formerly, he worked at the department of Quality Measures and Research at the Chief Phycian's Office of Clalit. He is a lecturer at Ben-Gurion University of the Negev, Beer Sheva, Israel.

**Yael Rosenbluth **is the Head of Information Services in the Chief Physician Office at Clalit Health Services Headquarters, and holds a Master Degree in Library & Information Studies.
